# A Dual‐Salt Gel Polymer Electrolyte with 3D Cross‐Linked Polymer Network for Dendrite‐Free Lithium Metal Batteries

**DOI:** 10.1002/advs.201800559

**Published:** 2018-07-13

**Authors:** Wei Fan, Nian‐Wu Li, Xiuling Zhang, Shuyu Zhao, Ran Cao, Yingying Yin, Yi Xing, Jiaona Wang, Yu‐Guo Guo, Congju Li

**Affiliations:** ^1^ Beijing Institute of Nanoenergy and Nanosystems Chinese Academy of Sciences Beijing 100083 P. R. China; ^2^ School of Nanoscience and Technology University of Chinese Academy of Sciences Beijing 100049 P. R. China; ^3^ School of Energy and Environmental Engineering University of Science and Technology Beijing Beijing 100083 China; ^4^ School of Materials Science and Engineering Beijing Institute of Fashion Technology Beijing 100029 China; ^5^ Beijing Key Laboratory of Clothing Materials R&D and Assessment Beijing 100029 China; ^6^ CAS Key Laboratory of Molecular Nanostructure and Nanotechnology CAS Research/Education Center for Excellence in Molecular Sciences Institute of Chemistry Chinese Academy of Sciences (CAS) Beijing 100190 P. R. China

**Keywords:** cross‐linked polymers, gel electrolytes, lithium dendrites, lithium metal batteries

## Abstract

Lithium metal batteries show great potential in energy storage because of their high energy density. Nevertheless, building a stable solid electrolyte interphase (SEI) and restraining the dendrite growth are difficult to realize with traditional liquid electrolytes. Solid and gel electrolytes are considered promising candidates to restrain the dendrites growth, while they are still limited by low ionic conductivity and incompatible interphases. Herein, a dual‐salt (LiTFSI‐LiPF_6_) gel polymer electrolyte (GPE) with 3D cross‐linked polymer network is designed to address these issues. By introducing a dual salt in 3D structure fabricated using an in situ polymerization method, the 3D‐GPE exhibits a high ionic conductivity (0.56 mS cm^−1^ at room temperature) and builds a robust and conductive SEI on the lithium metal surface. Consequently, the Li metal batteries using 3D‐GPE can markedly reduce the dendrite growth and achieve 87.93% capacity retention after cycling for 300 cycles. This work demonstrates a promising method to design electrolytes for lithium metal batteries.

Energy crisis is confusing human's daily life nowadays, appealing much attention to energy generation and energy storage. Of all the energy‐saving devices, rechargeable batteries are playing more and more important roles in energy storage devices and portable electronic vehicles.^1^, ^2^, ^3^, ^4^ Lithium metal is highly rated due to its high theoretical capacity of 3860 mAh g^−1^, compared with graphite anodes (372 mAh g^−1^), extremely low redox potential (−3.04 V vs SHE), and low density (0.534 g cm^−3^). Thus, lithium metal battery (LMB) is expected to be applied in numerous circumstances because of its intrinsic merit of high energy density.^5^ Although such superiorities mentioned above have attracted extensive researchers' interests, lithium dendrites generated on lithium metal severely restrained the practical usages of LMB.^4^, ^6^ Lithium metal is relatively unstable concerning thermodynamics with regard to traditional liquid electrolytes, regretfully. Volume expansion results from lithium metal plating‐stripping processes usually get solid electrolyte interphase (SEI) cracks, inducing the exposure of fresh lithium metal and subsequent side reactions. Consequently, liquid electrolyte could hardly inhibit the formation of lithium dendrites, which may cause short circuit of battery and affect performances. Therefore, series of substantial improvements are urgently needed to solve these critical issues.^7^, ^8^, ^9^, ^10^, ^11^, ^12^ Researchers began to focus on gel or solid‐state electrolytes, which showed their potential and possibility in uniform deposition of lithium, stable SEI layer formation, together with nontoxicity, nonflammability, and no leakage.^13^, ^14^

Conventional inorganic solid electrolyte could effectively restrain lithium dendrites. However, brittleness results in high fracture energy, and interphase contacting problems between electrolyte and lithium metal restrict practical application severely.^15^, ^16^, ^17^, ^18^, ^19^, ^20^ In comparison, polymer electrolyte improves the contact matters because of its flexibility, but the low ion conductivity under room temperature and narrow electrochemical window greatly impose restrictions on its further applications. Hence, in order to possibly solve above mentioned issues, gel polymer electrolyte (GPE) with relatively higher ion conductivity is expected to be an appropriate candidate to realize the uniform deposition of lithium metal, forming a robust SEI layer above lithium metal surface.^21^, ^22^, ^23^, ^24^, ^25^, ^26^, ^27^ Additionally, in order to improve ion conductivity of gel electrolytes, multifunctional polymers were employed by researchers to form rigid‐flexible cross‐linked network structures.^28^, ^29^, ^30^, ^31^ Still, corresponding reports emphasized that dual lithium salts system is proved to be valid for stabilizing interphases, enhancing ion conductivity, and further reducing the capacity loss in the long run.^32^, ^33^

Here, a novel dual‐salt (LiTFSI‐LiPF_6_) GPE with 3D cross‐linked polymer network was designed to reduce the dendrite growth and build stable SEI layers. The cross‐linked 3D polymerized by poly(ethylene glycol) diacrylate (PEGDA) and ethoxylated trimethylolpropane triacrylate (ETPTA) was produced simultaneously introducing dual‐salt electrolyte in the 3D structure, thereby enhancing thermostabilization and improving ion transference of gel electrolyte. The introduction of dual salt improves the ion conductivity and enhances the stability of SEI. With these advantages, the GPE shows an excellent performance in lithium dendrites blocking, and the capacity of LiFePO_4_|GPE|Li cell keeps 87.93% retention after 300 cycles, further furnishes a desirable reference for electrolyte designing of energy storage devices.

In this work, the 3D cross‐linked GPE was copolymerized by PEGDA and ETPTA through thermal initiated method. Multiple reaction sites of PEGDA and ETPTA provide possibilities of polymerizing reactions under thermal initiation, and the autopolymerization as well as copolymerization further forming a 3D cross‐linked structure. The specific synthesis route is illustrated in detail in **Figure** **1**. PEGDA and ETPTA are sequentially added together at a certain volume ratio (4:1 is the optimized ratio based on experiments). Compared with irregular lithium deposition of liquid electrolyte, the tight compact of GPE with uniform Li‐ion distribution ensures the uniform deposition of lithium, results in lithium dendrite restraint. Apparently, the in situ synthesis way we adopt not only simplifies the assembly process of the LMBs greatly,^34^ but also improves the contact issues of anode and electrolyte compared with other traditional solid electrolytes.^35^

**Figure 1:**
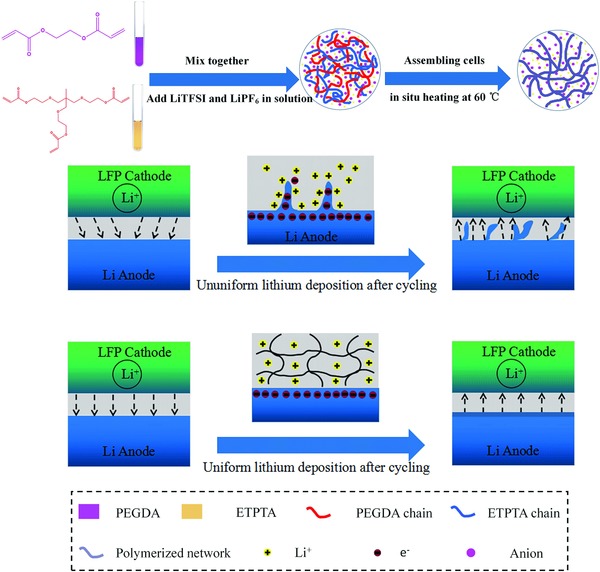
Step process for in situ polymerization of GPE.

In order to demonstrate the cross‐link reaction and the products after polymerization, several test methods are applied in this experiment. The scanning electron microscopy (SEM) is used to observe morphology of GPEs. As shown in **Figure** **2**a, cellulose nonwoven is composed of random nanofibers with large‐sized pores, which provide networks for movement of chains and also function as mechanical supporting framework. The surface morphology of polymerized GPE is shown in Figure 2b; it reveals that a homogeneous and smooth membrane is obtained after polymerization, and it also demonstrates that polymer matrix and Li salt are all uniformly filled in the pores of fibers. The cross‐section image is embedded in Figure 2b, which shows that the thickness of GPE is ≈100 µm. The X‐ray diffraction (XRD) is used to measure the crystallinity of the structures (Figure 2c), it distinctly shows that all the gel electrolytes, whatever volume ratio it is, are totally amorphous and lithium salts are absolutely dissolved in the mixture, which are beneficial to Li‐ion transfer and further enhance the ion conductivity of the GPEs. LiTFSI particles added in this system not only form as plasticizer to inhibit polymer crystalline, but form as one part of dual lithium system.

**Figure 2:**
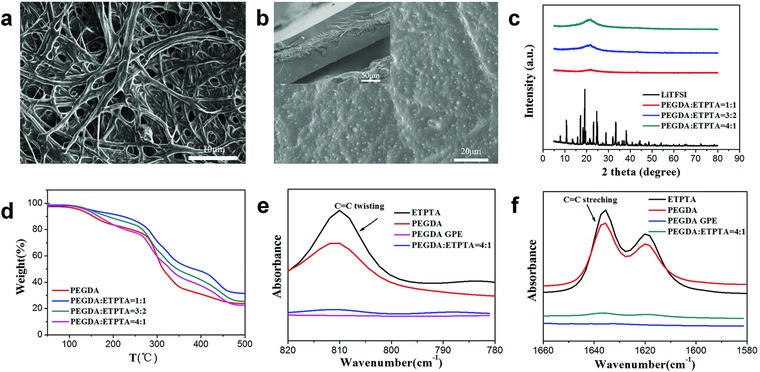
Characterization of composition and phases of GPE: a) SEM image of the surface of cellulose membrane; b) SEM image of the surface of GPE; c) XRD patterns of pure PEGDA GPE and different ratio PEGDA‐*co*‐ETPTA GPEs; d) TGA thermograms of pure PEGDA GPE and different ratio PEGDA‐*co*‐ETPTA GPEs; e,f) FTIR spectra of enlarged regions of PEGDA‐*co*‐ETPTA GPE at the ratio of 4:1.

The thermogravimetric analysis (TGA) thermograms are shown in Figure 2d, to identify the thermal stability of GPEs. It could be clearly seen that GPEs show negligible weight loss until temperature reaches up to 280 °C. The nonlinear changing relationship of weight loss and temperature verifies nonuniform polymerization of monomers, and the formation of 3D cross‐linked structure. Thermal behaviors are experimented to confirm the safety of GPE. It can be vividly seen that our prepared GPE possesses a relatively low flammability and is resistance to flame (Figure S1a, Supporting Information), compared with commercial separator with liquid electrolyte (Figure S1b, Supporting Information). In addition, GPE and commercial separator are both heated at 150 °C for 10 min. It could be clearly seen that commercial separator suffers a severe shape shrinkage (Figure S1c, Supporting Information), while GPE shows ignorable shape change with the same condition (Figure S1d, Supporting Information). The phenomena reveal that the GPE can effectively impede the safety dangers caused by internal short circuit and improve the applications under high‐temperature circumstances.

Fourier‐transform infrared (FTIR) spectrum is used to illustrate the consequence of polymerization of two monomers. Figure 2e,f exhibits the enlarged regions of the infrared spectra, typical peaks are corresponding to C=C bonds of the acrylate end groups in both PEGDA and ETPTA. The IR peaks in the range of 780–830 cm^−1^ are mainly ascribed to C=C twisting vibration of the acrylate groups, while the peaks in the range of 1610–1680 cm^−1^ are contributed from C=C stretching vibration of the acrylate groups. Furthermore, the characteristic peaks disappeared after polymerization compared with monomers, demonstrating the complete cross‐linking of the monomers, forming a 3D cross‐linked framework.

Energy‐dispersive X‐ray spectroscopy (EDS) is used to detect and track element dispersion and interconnectedness of GPE. As shown in Figure S2b,c (Supporting Information), EDS maps represent the information of P‐K and S‐K revealing the distribution and proportion of LiTFSI and LiPF_6_ separately. It clearly demonstrates that both LiPF_6_ and LiTFSI are uniformly dispersed in the system. And the above results are in accordance with the previous experiment.

Linear sweep voltammetry (LSV) is used to demonstrate the electrochemical stability of differently produced GPEs at 25 °C in **Figure** **3**a. All the GPEs composed of PEGDA and ETPTA are distinctly stable up to 4.7 V versus Li^+^|Li, compared with PEGDA‐based GPE (4.2 V vs Li^+^|Li), which makes it suitable for application in higher voltage batteries. The NCM|Li battery in the voltage range of 3.0–4.3V is assembled to confirm its electrochemical stability (Figure S3, Supporting Information). As a prerequisite parameter of electrolyte and a nonnegligible factor of the achievement of high power output capability, *t*_Li+_ is another important way to evaluate the electrochemical ability of electrolytes (Figure 3b). Compared with conventional liquid electrolyte and other polymer electrolytes which mainly ranges from ≈0.2 to 0.4. The *t*_Li_^+^ of our designed GPE reaches up to 0.72, which exceeds most gel electrolytes, hence decrease the concentration gradients at electrode surfaces and enable achieving a high power capability. In order to measure the dynamic stability, a polarization test is also tested by using Li|GPE|Li symmetric cells (Figure S4, Supporting Information). It can be seen that GPE exhibits a relatively steady cycling performance with lithium metal plating and stripping in Li|GPE|Li while under a constant current density of 0.5 mA cm^−2^. In comparison, liquid electrolyte symmetric cell shows irregular voltage changes at 350 h. And the consequence verifies that a relatively more stable interphase is formed in GPE cells.

**Figure 3:**
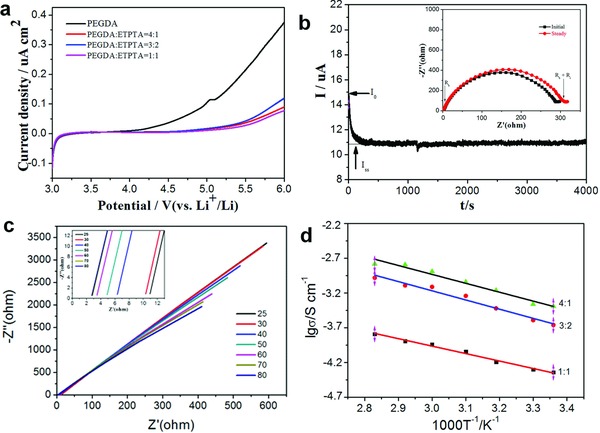
Electrochemical properties of GPE: a) Linear sweep voltammetry of different GPEs; b) current variation with polarization of a Li|GPE|Li symmetrical cell with an applied potential of 50 mV and EIS before and after polarization; c) EIS of a Li|GPE|SS symmetrical cell with the elevation of temperature; d) EIS of a Li|GPE|SS symmetrical cell of different volume ratio.

AC impedance techniques are used to assess the ion‐conducting properties of GPEs. As can be seen in Figure 3c, with the increase of the temperature, the ionic conductivity increases simultaneously. Showing 5.6 × 10^−4^ S cm^−1^ at 25 °C and 2.8 × 10^−3^ S cm^−1^ at 80 °C, indicating that higher temperature accelerates the movement of Li‐ion, revealing a temperature dependence. In addition, activation energy is illustrated to reflect the reaction difficulties, linear relationship of σ and temperature of GPE conform to typical Arrhenius‐type behavior with an *E*_a_ equal to 4.80 × 10^−2^ eV after calculation, which is extremely close to liquid electrolyte with commercial separator (4.20 × 10^−2^ eV). Moreover, the proportion of PEGDA is also another factor that affects the ion‐conducting properties. It can be distinctly distinguished from Figure 3d that a higher ion conductivity can be obtained with more addition of PEGDA, which demonstrates that the linear chain motion of PEGDA is beneficial to the transference of Li‐ion. Two main parts contribute to ion conductivity together. For one thing, the motion of PEGDA polymer chains greatly contributes to ion conductivity. For another, lithium ions are transferring through the complex dissociation of Li^+^ with O=C—O.^9^ It has been confirmed that C=O groups enable migration of Li^+^ faster than C—O—C. Ionic conductivities of different constituent copolymers are summarized in Figure S5 (Supporting Information). As a supplementary, dual Li salts are applied in this system to improve ion conductivities of GPEs and stabilize the interphases. The experiment also illustrates this phenomenon, dual Li salts benefit to the conductivity of GPE, which shows promoted ion conductivity of 5.6 × 10^−4^ S cm^−1^, compared with single LiTFSI (1.6 × 10^−4^ S cm^−1^) and LiPF_6_ (1.2 × 10^−4^ S cm^−1^) in GPEs (Figure S6, Supporting Information). The reason mainly ascribes to following aspects. First of all, the dissociation capacity of single lithium salt has been improved by introducing another lithium salt, further influencing the dissociation balance reaction of single lithium salt, inducing the forward reaction and increment of Li^+^.^36^, ^37^ As regard to Li^+^ transference, 3D polymerized network restrained the motion of TFSI^−^ and PF_6_^−^, while making available the pass through of Li^+^ due to volume effect. Thus, the dual Li salt 3D cross‐linked GPE reveals relative excellent performance on Li^+^ transference number and ionic conductivity. Moreover, according to previous research, LiPF_6_ in dual lithium salts could greatly stabilize Al foil and maintains electrical connection with the active materials.^33^

The aging stability is measured in the symmetrically nonblocking Li|GPE|Li cell by AC impedance method. It is shown in Figure S7 (Supporting Information) that the interfacial resistance approximately keeps steady with the evolution of time, which demonstrates that the GPE designed by us formed a stable interphase, little side reactions occur compared with liquid electrolytes.

In order to reveal the applications and electrochemical performances of the designed GPEs, LiFePO_4_|GPE|Li cells are fabricated by in situ synthesis. Furthermore, aiming to investigate the effect of the addition of ETPTA monomers in GPE, individual PEGDA‐based GPE is also experimented as a comparison. Setting the interval of voltage between 2.0 and 4.0V, it can be clearly seen from **Figure** **4**a that the addition of ETPTA greatly enhances the capacity of batteries under higher current densities. While PEGDA plays a more important role under lower current densities, as presented in Figure S8 (Supporting Information), the LiFePO_4_|GPE|Li cell delivered a discharge capacity of 137 mAh g^−1^ for the first cycle, and keeps 136 mAh g^−1^ after 50 cycles at a rate of 0.2 C, with the capacity retention of 98.5%. Of all the mixtures, PEGDA:ETPTA = 4:1 reveals relatively the best performances under varied current densities. The charge–discharge curves of batteries under this mixture ratio are revealed in Figure 4b, which delivers reversible capacities of 135, 133, 127, and 103 mAh g^−1^ at 0.1, 0.2, 0.5, and 1 C, respectively. The cycling performance is shown in Figure 4c, the capacity of LiFePO_4_|GPE|Li cell has an initial discharge capacity of 116.9 mAh g^−1^ at 0.5 C, and retaining 87.93% after 300 cycles. The higher capacity obtained with the addition of ETPTA mainly ascribes to the enhanced electrochemical stability as well as functionalized triple‐branch structure, which applies more networks for transference of lithium ions. While PEGDA contributes more to capacity under lower current density due to its linear structure. In order to further verify the difference of different ratio performances, the interfacial resistance and reversibility of GPEs are evaluated by electron impedance spectroscopy (EIS), which is shown in Figure 4d. It demonstrates that with the increment of ETPTA, the impedance decreases simultaneously, further explaining the better performances of batteries under higher current densities.

**Figure 4:**
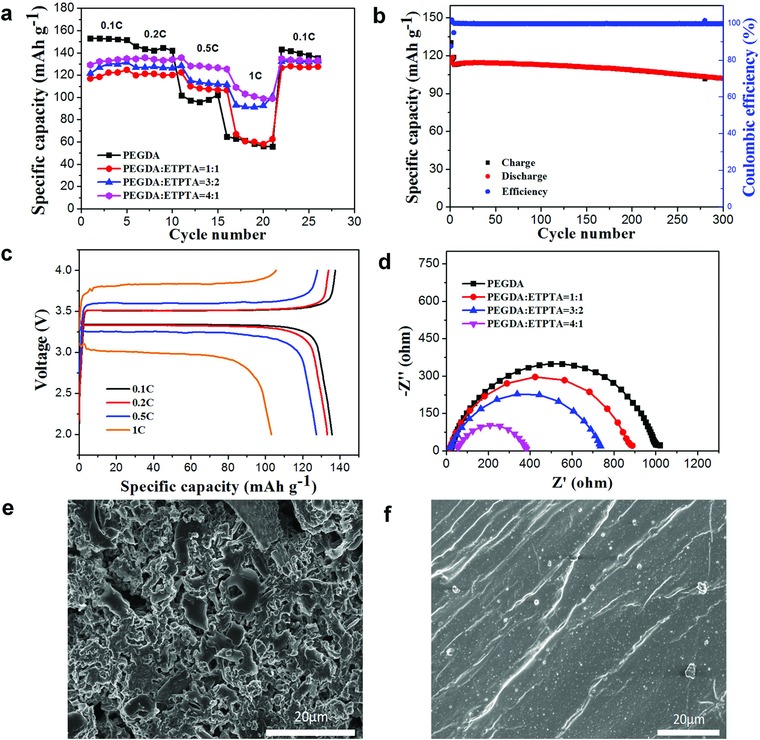
Electrochemical performance of GPE: a) Rate capability of LiFePO_4_|Li cell using PEGDA GPE and different ratio GPEs; b) rate performance of PEGDA‐*co*‐ETPTA GPE with 4:1 (v:v); c) cycling performance of LiFePO_4_|Li cell using PEGDA:ETPTA = 4:1 (v:v) GPE at 0.5 C at 20 °C; d) EIS of Li|GPE|Li of different GPEs; e) the Li electrode obtained from a LiFePO_4_|separator liquid electrolyte|Li cell and f) from a LiFePO_4_|GPE|Li cell after 100 cycles at 0.5 C.

The surface morphology changes before and after 0.5 C for 100 cycles of the lithium anode have been obtained through SEM. The fresh lithium anode, lithium anode after cycling with liquid electrolyte and GPE are compared to demonstrate the lithium dendrite formation. As presented in **Figure** **5**c, the fresh lithium anode without any reaction is smooth and dense. However, a quantity of dendrites could be clearly observed on the lithium anode surface with liquid electrolyte after cycling at 0.5 C for 100 cycles (Figure 4e). On the contrary, little apparent lithium dendrites emerged on the surface of lithium anode with GPE (Figure 4f), which is experimented in the same circumstance. These results reflect the function of GPE of restraining the formation of lithium dendrites compared with conventional liquid electrolyte, further demonstrating that GPEs could greatly improve the cycling performance of batteries and thus eliminating the dangers of short circuits caused by lithium dendrites.

**Figure 5:**
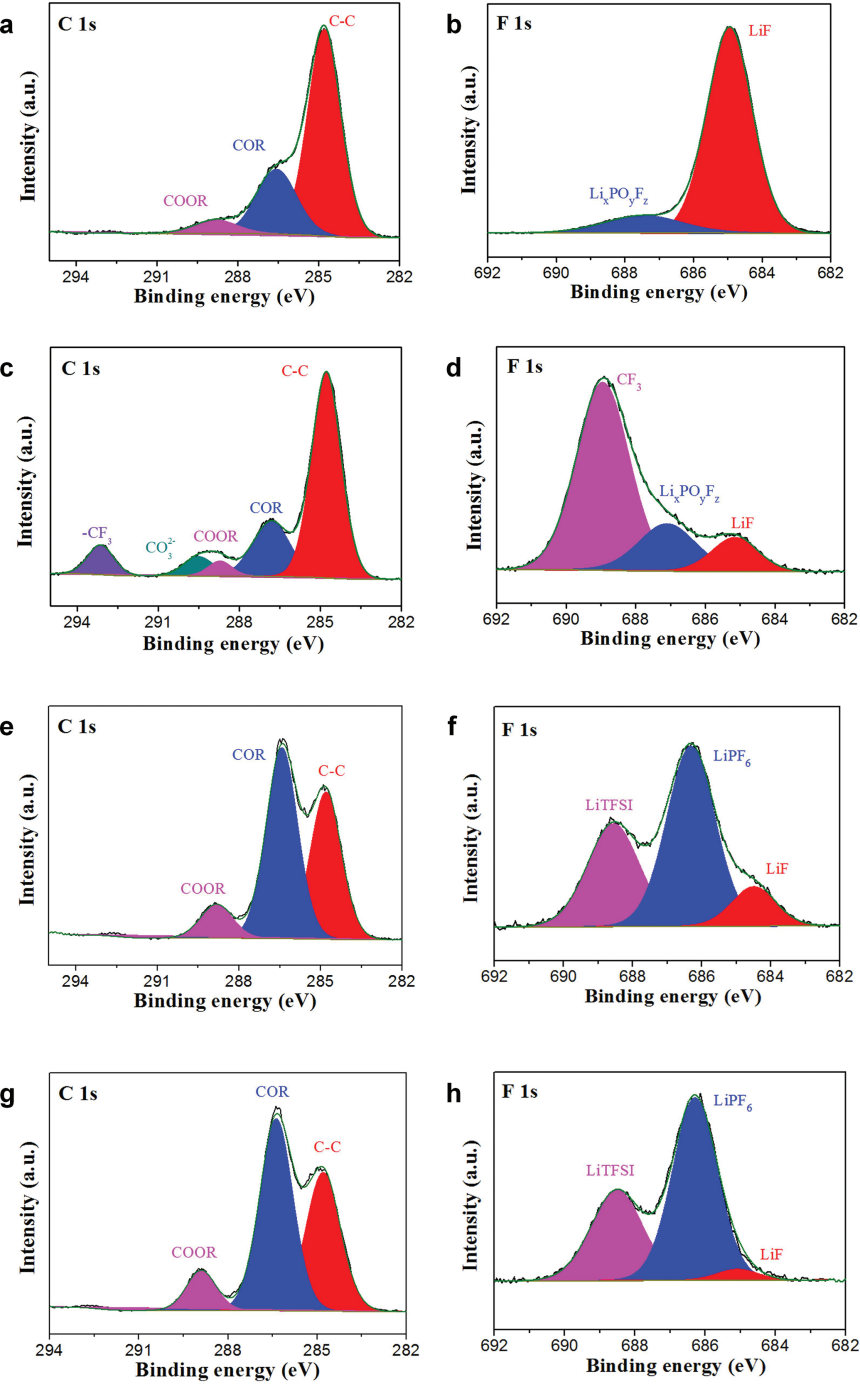
Characterization of the components of SEI produced on cycled lithium metal surface. a,b) XPS spectra of C 1s and F 1s for lithium metal retrieved from liquid electrolyte (LiPF_6_) without cycling; c,d) liquid electrolyte (LiPF_6_) after cycling for ten cycles; e,f) dual lithium salts GPE before cycling; g,h) dual lithium salts GPE after cycling for ten cycles.

X‐ray photoelectron spectroscopy (XPS) is employed to investigate the composition and valence of SEI layer during cycling in Li|LiFePO_4_ batteries. For the Li metal anode in liquid electrolyte, the COR (286.6 eV)^38^, ^39^ and COOR (288.8 eV)^38^, ^39^ groups (Figure 5a) emerge during the SEI formation. After cycling, there are a lot of CO_3_^2−^ (289.5 eV)^36^, ^40^ and CF_3_ (293.1 eV)^38^, ^39^ groups (Figure 5c) on the surface of Li metal anode because of the decomposition of carbonate solvents and Li salt anions. Seen from the F 1s spectra (Figure 5b), the LiF (684.9 eV) and Li*_x_*PO*_y_*F*_z_* (687.4 eV)^41^, ^42^ can be observed during the SEI formation, which are the decomposition products of Li salt anions. After cycling, the amount of LiF and Li*_x_*PO*_y_*F*_z_* change significantly and a lot of CF_3_ (689.1 eV) groups emerge (Figure 5d), indicating the decomposition of LiTFSI and LiPF_6_ during the cycling.^38^, ^43^ Consequently, in the LMB using liquid electrolyte, the reduction products of electrolyte markedly increase in the SEI layer after cycling, elucidating the SEI layer is too fragile to protect Li metal anode.

For the Li|LiFePO_4_ battery using cross‐linked network‐GPE, a large amount of COR and COOR groups (Figure 5e) are found on the surface of Li metal anode, corresponding to —CH_2_—O and —CHOO— groups in the cross‐linked network structure, respectively.^44^ The ratio of COR and COOR agrees with the ratio of —CH_2_—O and —CHOO— groups approximately in the PEGDA and ETPEA mixture. These groups are stable and the reduction products (CF_3_, CO_3_^2−^) of electrolyte cannot be detected after cycling (Figure 5g). Seen from the F 1s spectra (Figure 5f), the peaks at 686.3 and 688.5 eV are assigned to the LiPF_6_ and LiTFSI, respectively. Except for a little change in LiF, no obvious change in the surface composition of Li metal anode is evident after cycling. In the LMB using GPE, the SEI layer of Li metal anode is constituted by cross‐linked network structure and GPE, and this interphase structure is stable during cycling. Therefore, the cross‐linked network structure with GPE builds a robust and conductive SEI layer on the surface of Li metal anode, which is beneficial to the dendrite growth suppression and long‐life cycling.

In summary, a dual‐salt (LiTFSI‐LiPF_6_) GPE with 3D cross‐linked polymer network is successfully designed by in situ polymerization of PEGDA and ETPTA. A high ionic conductivity (0.56 mS cm^−1^ at room temperature) is obtained simultaneously with a robust and conductive SEI on the lithium metal surface, which achieves 87.93% capacity retention after cycling for 300 cycles of battery and uniform lithium deposition. Additionally, lithium dendrites are effectively restrained by this 3D‐GPE. The functional monomers are applied in work function as follows: the linear molecular chains of PEGDA greatly benefit the lithium ions transference, while triple branches of ETPTA largely acting as a cross‐linking structure and forming networks. Consequently, the GPE basically solves the existing intractable issues of lithium metal batteries, ensuring an enhanced ionic conductivity and blocking lithium dendrite effective. Available GPE with simple in situ synthesis method and excellent performance make it possible for further applications and offers a reference to subsequent energy storage research.

## Experimental Section

*Synthesis of Gel Polymer Electrolyte*: PEGDA, ETPTA, and AIBN in this work were all purchased from Aladdin. PEGDA‐*co*‐ETPTA polymer gel electrolyte was prepared through in situ thermal polymerization method. ETPTA and PEGDA were added into a bottle as a precursor at different volume ratio. Then LiTFSI particles and LiPF_6_ in nonaqueous solution (EC:DMC:DEC = 1:1:1) were added at the mole ratio of 1:1, with magnetic stirring for 30 min. Finally, when all the additions were uniformly distributed into the solution, 1 wt% AIBN as the thermal initiator was added into the as‐prepared solution with continuous magnetic stirring for another 30 min. Mixed solution was injected into cell and assembled in glove box after stirring. Then, the cells were heated at 60 °C for 3 h. All procedures for preparing the polymer gel electrolyte were operated in an Ar‐filled glove box with the concentrations of moisture and oxygen below 0.01 ppm.

*Structural Characterization of GPE*: The morphologies and characteristics of the GPEs were characterized by the following methods and equipment. The morphologies of GPEs and Li metal foil were observed by a SEM (SU8020) at 5 kV, 10 µA. The crystal structure was characterized by XRD with Cu Kα radiation. A FTIR test was conducted on a spectrometer (Vertex 80, Bruker) in the frequency range of 400–4000 cm^−1^ with a resolution of 4 cm^−1^ and 32 scans at room temperature. TG were tested under a flow of nitrogen at the rate of 10 °C min^−1^, with temperature stabilized at 50 °C for 30 min. EDS was applied to analyze different elements of GPE by SU 8020. XPS was used to demonstrate different output products.

*Electrochemical Analysis of GPE*: The galvanostatic charging–discharging of battery cells was carried out by the commercial battery testing system (LAND CT2001A). AC impedances (from 100 kHz to 0.01 Hz) of the batteries were performed by an electrochemical workstation (CHI660E). The electrochemical stability window of GPE was measured by LSV at a scan rate of 1.0 mV s^−1^.

The calculation of *t*_Li+_ resulted from Bruce–Vincent–Evans equation as follows

(1) $$t_{\mathrm{Li}}{}^{+}=\frac{I_{\mathrm{ss}}(\Delta V-I_{o}R_{o})}{I_{o}(\Delta V-I_{\mathrm{ss}}R_{\mathrm{ss}})}$$

Δ*V* is the applied polarization voltage (Δ*V* = 50 mV), *I*_0_ and *R*_0_ are the initial current and interfacial resistance before polarization, respectively, and *I*_ss_ and *R*_ss_ are the steady‐state current and interfacial resistance after polarization for 4000 s, respectively.

The calculation of σ is calculated by the following equation

(2) σ=d/RS

where *d* is the thickness of electrolyte, *R* is the interfacial resistance, and *S* is the area of electrolyte.

Activation energy *E*_a_ was used to demonstrate the difficulty level of Li‐ion transference. The behavior of σ and *E*_a_ mainly obeys the Arrhenius equation

(3) $$\sigma =\sigma_{0}\exp\left(-E_{a}/RT\right)$$

For the LFP|Li test, a slurry of LFP, super‐P, and poly(vinylidene fluoride) dissolved in N‐methly‐2‐pyrrolidone was cast onto an aluminum foil. The cast film was dried in a vacuum oven at 120 °C for 720 min. The active material weight of cathode applied in this experiment is ≈1.2 mg cm^−2^.

## Conflict of Interest

The authors declare no conflict of interest.

## Supporting information

Supplementary
