# Supplementary material for: A Dual‐Salt Gel Polymer Electrolyte with 3D Cross‐Linked Polymer Network for Dendrite‐Free Lithium Metal Batteries
Source: Adv Sci (Weinh). 2018 Jul 13;5(9):1800559. doi: 10.1002/advs.201800559 (PMC6145227; doi:10.1002/advs.201800559)
Supplement: Supplementary file 1 — Supplementary [file ADVS-5-1800559-s001.pdf]

Copyright WILEY-VCH Verlag GmbH & Co. KGaA, 69469 Weinheim, Germany, 2018.

ADVANCED SCIENCE
Open Access

Supporting Information

for *Adv. Sci.,* DOI: 10.1002/advs.201800559

A Dual-Salt Gel Polymer Electrolyte with 3D Cross-Linked Polymer Network for Dendrite-Free Lithium Metal Batteries

Wei Fan, Nian-Wu Li, Xiuling Zhang, Shuyu Zhao, Ran Cao, Yingying Yin, Yi Xing, Jiaona Wang, Yu-Guo Guo, and Congju Li**

Copyright WILEY-VCH Verlag GmbH & Co. KGaA, 69469 Weinheim, Germany, 2016.

Supporting Information

A Dual-Salt Gel Polymer Electrolyte with 3D cross-linked Polymer Network for Dendrite-Free Lithium Metal Batteries

Wei Fan[1,⊥], *Nian-Wu Li*[1,⊥], *Xiuling Zhang*[1,⊥], *Shuyu Zhao*[2], *Ran Cao*[1], *Yingying Yin*[1], *Yi Xing*[2], *Jiaona Wang*[4], *Yu-Guo Guo*[3,]*, *Congju Li*[1,2,]*

Supporting information

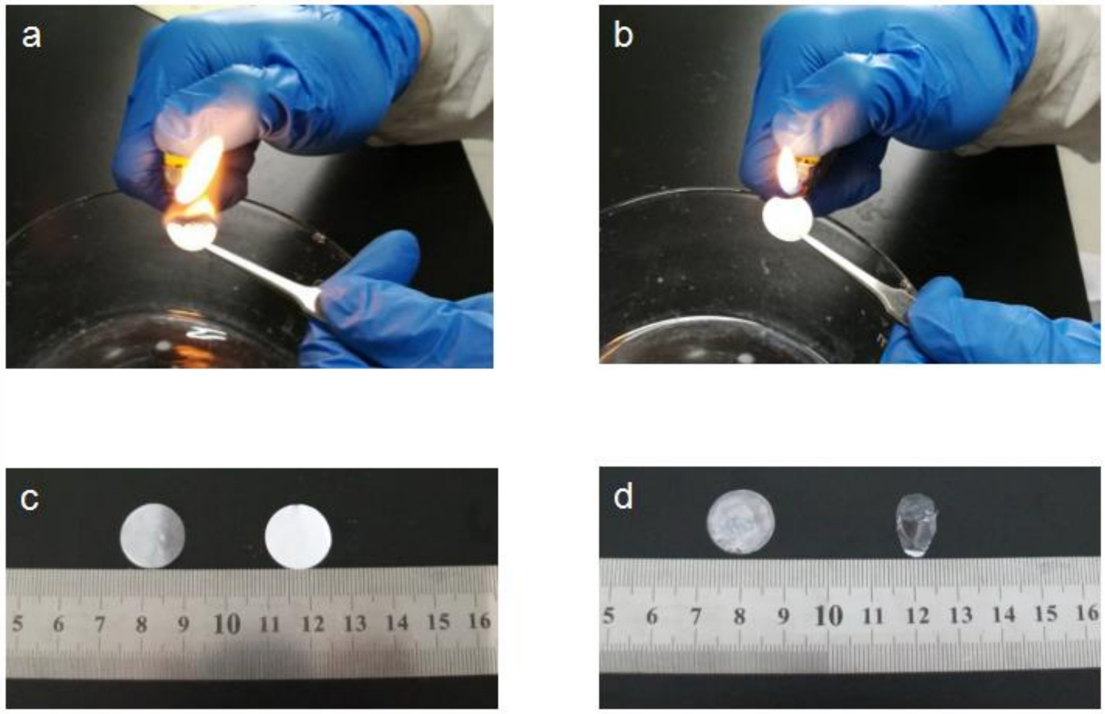

Figure S1. Combustion testing of a) Commercial PE separator, b) PGE membrane. Comparison photographs of the thermal shrinkage of the 3D cross-linking membrane (left) and a commercial separator (right) c)before and d) after exposure to 150 ℃ for 10 min.

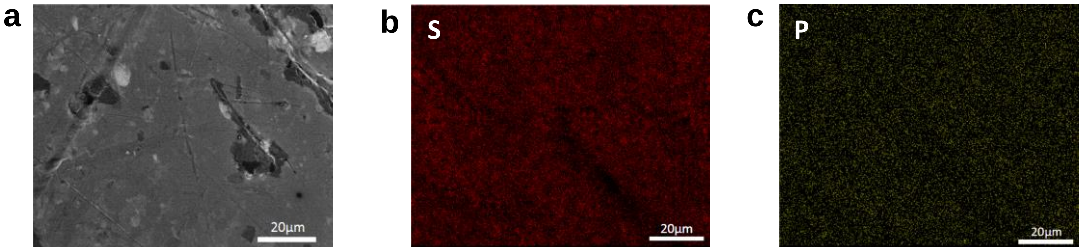

Figure S2. a) SEM of morphology of GPE; b) EDS map of P-K; c) S-K of surface section.

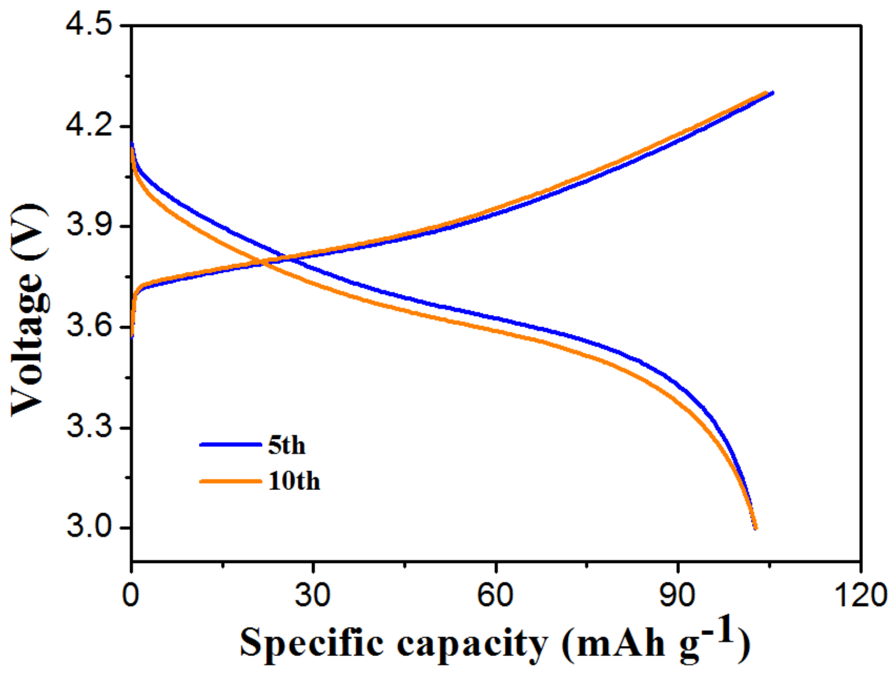

Figure S3. Charging-Discharging curves of the fifth and tenth cycle of Li|NCM at 0.5C at 25 ^{o}C. The charging-discharging range is 3.0V-4.3V.

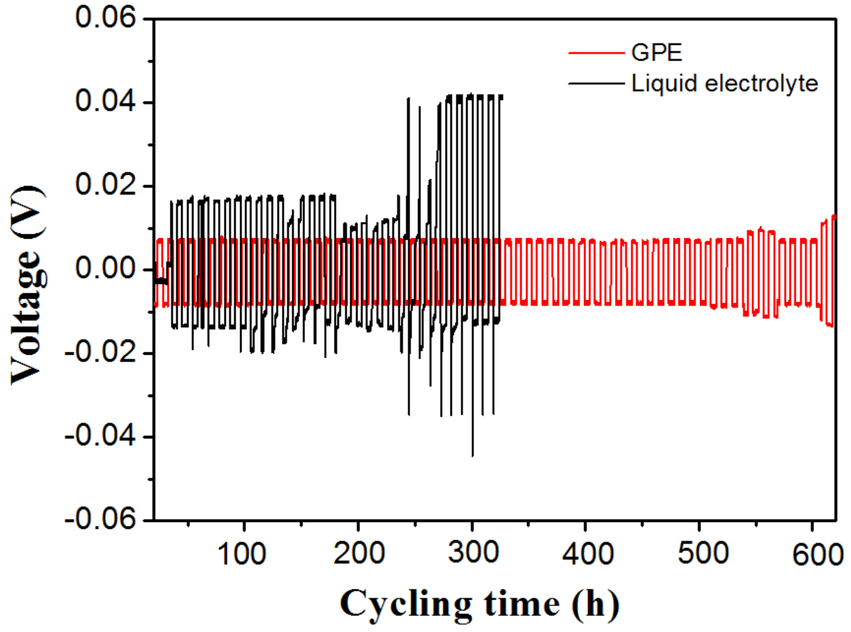

Figure S4. Polarization test by using Li|GPE|Li symmetric cells and liquid electrolyte

	Ionic conductivity (mS/cm)
**PEGDA:ETPTA=1:1**	0.04
**PEGDA:ETPTA=3:2**	0.25
**PEGDA:ETPTA=4:1**	0.56
**ETPTA**	0.18

Figure S5. Ionic conductivity of different polymer constitution.

	$LiPF_6$	LiTFSI	Dual Li salts
**Ionic conductivity (mS/cm)**	0.12	0.16	0.56

Figure S6. Ionic onductivity of different Li salt constitution.

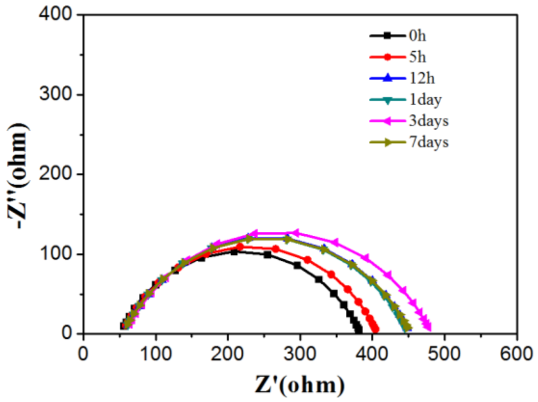

Figure S7. Impedance response with time evolution of the Li|GPE|Li.

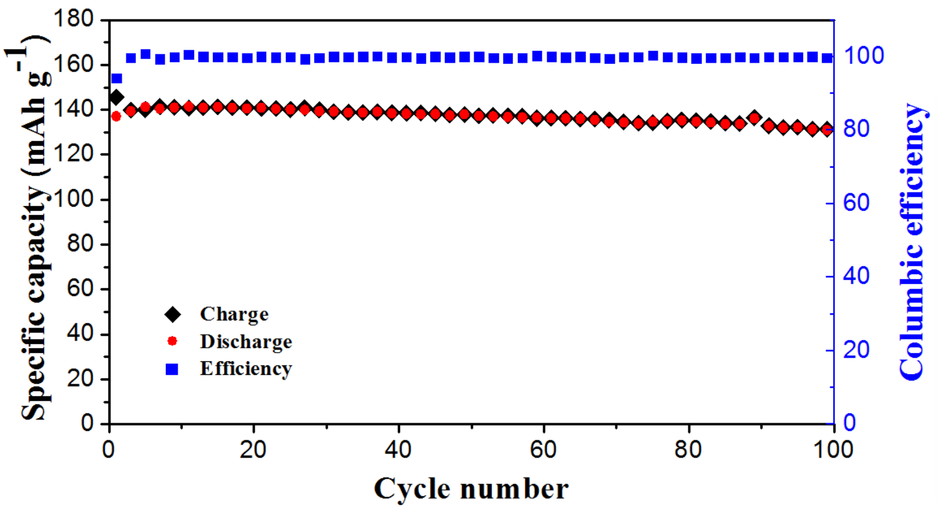

Figure S8. Battery performance of PEGDA based GPEs at the rate of 0.2 C at 20 °C.

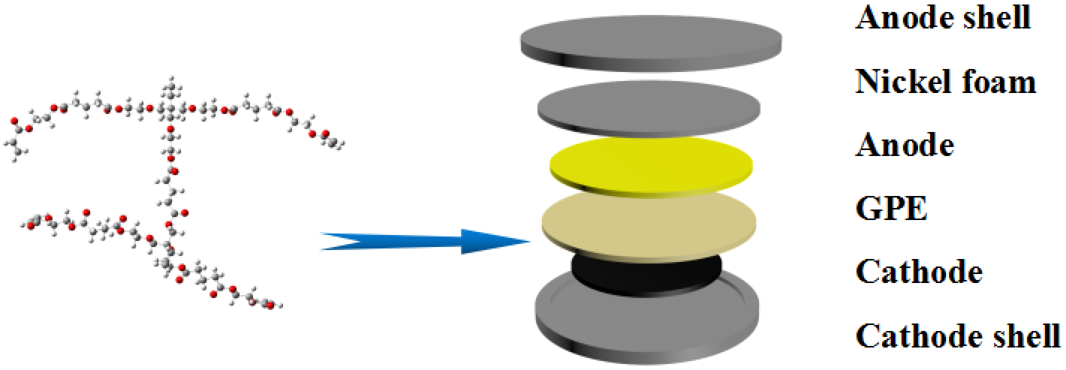

Figure S9. Structural illustration of GPE battery.

	Thermal safety (°C)	Ion Conductivity(mS/cm)	Li^+ transference
**PVA-CN solid electrolyte**[1]	160	0.3 at room temperature	0.57
**PPC polymer electrolyte**[2]	-	0.3 at 20°C	-
**PINs**[3]	250	0.532 at 20°C	-
**PVDF/PEO SPE**[4]	158	0.303 at room temperature	-
**PVDF/HEC/PVDF**[5]	290	0.88 at room temperature	0.57
**TPU/PEO SPE**[6]	314	0.53 at 60°C	0.31
**PEGDA-ETPTA GPE**	280	0.56 at room temperature	0.72

Figure S10. Physical and electrochemical properties of our produced GPE and other GPEs.

References

[1] Zhou, D.; He, Y.-B.; Liu, R.; Liu, M.; Du, H.; Li, B.; Cai, Q.; Yang, Q.-H.; Kang, F. Adv. Energy Mater. **2015**, 5, (15), 1500353.

[2] Zhang, J.; Zhao, J.; Yue, L.; Wang, Q.; Chai, J.; Liu, Z.; Zhou, X.; Li, H.; Guo, Y.; Cui, G.; Chen, L. Adv. Energy Mater. **2015**, 5, (24), 1501082.

[3] Zhang, P.; Li, M.; Yang, B.; Fang, Y.; Jiang, X.; Veith, G. M.; Sun, X. G.; Dai, S. Adv. Mater. **2015**, 27, (48), 8088-94.

[4] Deng, F.; Wang, X.; He, D.; Hu, J.; Gong, C.; Ye, Y. S.; Xie, X.; Xue, Z. J. Membr. Sci. **2015**, 491, 82-89.

[5] Zhang, M. Y.; Li, M. X.; Chang, Z.; Wang, Y. F.; Gao, J.; Zhu, Y. S.; Wu, Y. P.; Huang, W. Electrochim. Acta **2017**, 245, 752-759.

[6] Tao, C.; Gao, M.-H.; Yin, B.-H.; Li, B.; Huang, Y.-P.; Xu, G.; Bao, J.-J. Electrochim. Acta **2017**, 257, 31-39.
